# Supplementary material for: Linking Indices for Biodiversity Monitoring to Extinction Risk Theory
Source: Conserv Biol. 2014 May 12;28(6):1575–83. doi: 10.1111/cobi.12308 (PMC4253318; doi:10.1111/cobi.12308)
Supplement: Supplementary file 1 — Supplementary [file cobi0028-1575-SD1.pdf]

Supporting Information

Linking indices for biodiversity monitoring to extinction risk theory

Michael A. McCarthy[1], Alana L. Moore[1], Jochen Krauss[2], John W. Morgan[3], Christopher F. Clements[4]

[1]School of Botany, The University of Melbourne, Parkville, Victoria 3010, Australia

[2]Department of Animal Ecology and Tropical Biology, Biocentre, University of Würzburg, Am Hubland, 97074 Würzburg, Germany

[3]Department of Botany, La Trobe University, Bundoora, Victoria 3086, Australia

[4]Department of Animal and Plant Sciences, University of Sheffield, Sheffield, S10 2TN, UK

Appendix S1. Details on the datasets used for evaluating the indices

The abundances of butterfly species in 31 patches of habitat were estimated in both 1996 and 2000 (Krauss et al. 2003). Data were restricted to those species present in at least 24 of the 31 patches so that extinction risk was assessed for a pool of species that were relatively similar at all sites. Thus, differences among sites tended to reflect differences in abundance rather than inherent differences among species. For all these species, probabilities of detection given presence, based on occupancy/detection models (Stauffer et al. 2002) using data on detections in sets of 5-minute intervals, were estimated to be >95% in the surveys. In each patch, between 13 and 16 of the species being considered were observed in the first instance. For each index, we plotted the mean extinction risk (calculated as the proportion of species that went extinct) against the index. For this case study, the time horizon is relatively short, T = 8

(assuming two generations per year). For the second index, this results in $b \approx 6.6$ assuming $\sigma = 0.1$. Greater variability in the population dynamics (i.e., $\sigma > 0.1$) would lead to smaller values for b. The data were also analysed when the data were restricted to species that were genuine grassland specialists, but as the results were qualitatively the same, only results with the larger number of species are reported.

The composition and abundance of all native plant species of ten grassy woodland patches in western Victoria, Australia were surveyed in 2006 (Sutton & Morgan 2009). These data were compared to the species composition and population abundance of the same patches observed in 1975 to determine patch extinction rates. To reduce false absences, sites were surveyed on three occasions, with the time spent searching being proportional to each site's size and heterogeneity. To further minimize chances of missed detections, we restricted our analyses to perennial species. Finally, we limit variation in extinction risk due to idiosyncratic differences among species by considering only species that were present in at least eight of the ten patches in the analysis.

Abundances of the plants was estimated in 1975 using a four-point scale: "Very Rare" (less than two dozen individuals seen across the site), "Rare" (appearing in dozens), "Common" (appearing in hundreds), "Very Common" (appearing in thousands). The discretised and censored data meant we were unable to calculate the indices directly. Instead, we fitted a Pareto distribution to the abundance data, using maximum likelihood methods, and calculated the indices from the parameters of the estimated distribution. To fit the Pareto distribution, we assumed that the four abundance class were distinguished by threshold values of 24, 100 and 1000 (*i.e.*, "Very Rare" was assumed to be <24 individuals, "Rare" was 24-100, etc).

We chose to fit a Pareto distribution since, for the deterministic model with negative growth rate, if the mean time to extinction (T) is exponentially distributed with parameter θ,

abundance (x = exp[T/k]) is a Pareto random variable with scale parameter x_m = 1, and shape parameter $\alpha = k\theta$ (Krishnamorrthy 2006). We also fitted a log-normal distribution instead of a Pareto distribution. The results obtained were very similar in both cases, so we only present results for the Pareto. Assuming σ=0.1 and T=30 leads to b = 3.4 in the power mean index I_b.

Experimental protozoan communities were assembled with 4 ciliate species, at 2 temperatures (15 °C and 20 °C), and sampled for abundance data 3 times a week for 163 days (Clements et al. 2013). Four 3-species communities and one 4-species community were replicated at each temperature giving a total of 10 communities (a "community" in this case being a particular combination of species and temperatures). Extinction events were driven by either competitive exclusion or starvation. Population trends between days 5 and 9, abundance at day 9, and the proportion of species extinct by day 163, were calculated and averaged across replicates. Day 9 was chosen as the initial date because this day occurred prior to all but one extinction event, and effects of initial conditions on the community dynamics had attenuated. Each community was replicated 5 times each, except for one community in which a replicate was excluded when a species went extinct prior to day 9. The indices for each of the 10 communities were then compared to the proportion of species extinct by day 163. Given the ecological time frame of this experiment (≈200 protozoan generations; Clements et al. 2013), the extinctions observed indicate possible long-term extinction trends. The parameter b in the index based on the power mean was approximately 1.3, assuming σ = 0.1.

Appendix S2. Method for simulating expected correlation

Even if the probability of extinction of species in a community were perfectly correlated with a particular index of extinction, the observed proportion of species going extinct would vary randomly around the actual probability of extinction because there are a finite number of

species in each community. This level of variation will tend to increase as the number of species declines. The consequence of this variation is that the observed correlations between the indices and the proportion of species going extinct would be less than one even if the indices were perfectly correlated with the probability of extinction. We evaluated the distribution of measured correlations that would be expected under the assumption that the indices were perfectly correlated with extinction probability to examine whether the measured correlation coefficients for the datasets were smaller than would be expected.

The analysis was conducted by simulating extinction of species in each of the communities. For the indices based on the geometric mean (I_g) and the power mean (I_b), we assumed that the probability of extinction of species in each community (p) was proportional to the index for that community. That is, $p = bI_j$, where I_j is the particular index being examined. The constant of proportionality was set for each dataset such that the means of p and I_j, were the same as those in the dataset (i.e., $b = \bar{p} / \bar{I}_j$). For the index based on the trend, the index sometimes took negative values, so we could not use this approach. Instead, we set $p = a + bI_t$, with a and b estimated from linear regression of the observed relationship.

For each species in each community, we simulated extinction by randomly determining whether extinction occurred given the specified probability of extinction p. That is, the number of extinctions, y, in each community was assumed to be drawn from a binomial distribution with parameters p and n, where n was the number of species originally in that community. The simulated proportion of species in each community that went extinction was then $\hat{p} = y/n$. For each dataset, we then calculated the correlation between $\hat{p}$ and I_j, which was recorded. This process was iterated 1 million times to generate a distribution of expected correlations under the assumption of perfect correlation between p and I_j given random variation in the observed values of p. The observed correlation coefficient can then be

compared to this distribution. If observed correlation coefficient lies outside the 95% prediction interval based on the simulated distribution of correlation coefficients, then it suggests the observed correlation is unexpected given the assumptions. Comparing the observed correlation to the simulated 95% interval is equivalent to testing a null hypothesis that the indices were perfectly correlated with extinction risk.

These comparisons indicate that the observed correlations are often within the anticipated range (Fig. S1). In the case of the index based on the power mean (I_b), the observed correlations are substantially smaller than expected for the butterfly and protozoan dataset. In the other cases, the observed correlations are generally consistent with the range of values that might be expected. The one other exception was the index based on the geometric for the butterfly dataset, where the observed correlation coefficient was actually larger than expected.

References for Supporting Information

Baillie, J. E. M., J. Griffiths, S. T. Turvey, J. Loh, and B. Collen. 2010. Evolution Lost: Status and Trends of the World's Vertebrates. Zoological Society of London, UK.

Clements, C. F., N. Worsfold, P. Warren, B. Collen, T. Blackburn, N. Clark, and O. L. Petchey. 2013. Experimentally testing an extinction estimator: Solow's Optimal Linear Estimation model. Journal of Animal Ecology **82**:345-354.

Krauss, J., I. Steffan-Dewenter, and T. Tscharntke. 2003. Local species immigration, extinction, and turnover of butterflies in relation to habitat area and habitat isolation. Oecologia **137**:591-602.

Krishnamoorthy, K. 2006. Handbook of statistical distributions with applications. Boca Raton, Chapman & Hall/CRC.

Stauffer, H. B., C. J. Ralph, and S. L. Miller. 2002. Incorporating detection uncertainty into presence-absence surveys for marbled murrelet. Pages 357-365 J. M. Scott, P. J. Heglund, M. L. Morrison, J. B. Haufler, M. G. Martin, W. A. Wall and F. B. Samson, editors. Predicting species occurrences: issues of accuracy and scale. : Island Press, Washtington, D.C.

Sutton, F. M., and J. W. Morgan. 2009. Functional traits and prior abundance explain native plant extirpation in a fragmented woodland landscape. Journal of Ecology **97**:718-727.

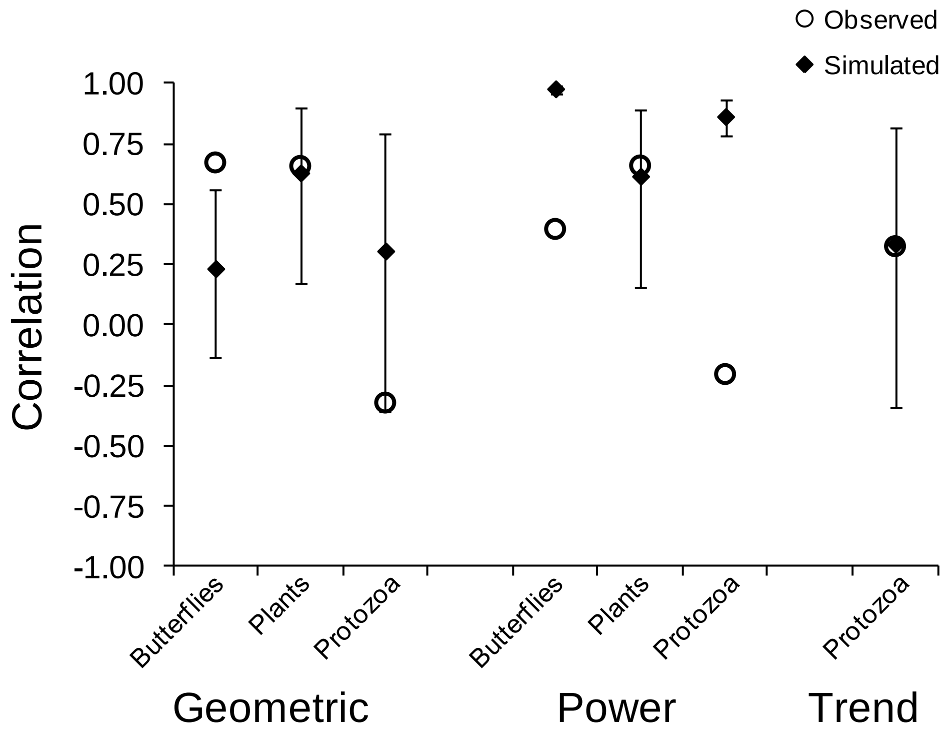

Fig. S1. The simulated distribution of correlation coefficients assuming that extinction probability is perfectly correlated with each index (diamond is the mean, and the bars are 95% prediction intervals) compared to the observed correlation (open circles).

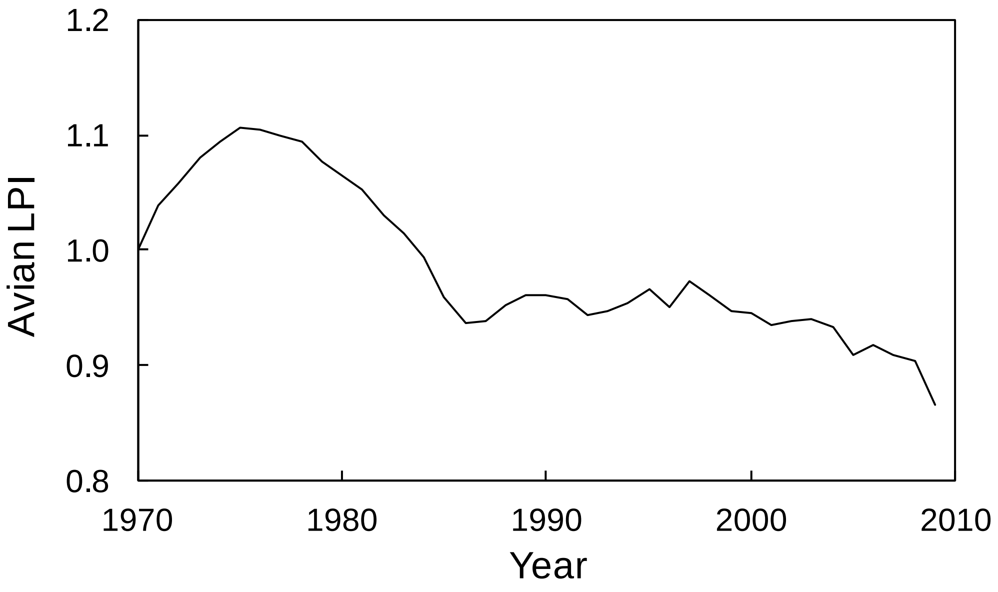

Fig. S2. The avian Living Planet Index (reproduced from Baille et al. 2010) that is the basis for Fig. 3.
